# Comparative Effectiveness of Bariatric Metabolic Surgery Versus Glucagon-Like Peptide-1 Receptor Agonists on Cardiovascular Outcomes and Mortality: A Meta-Analysis

**DOI:** 10.7759/cureus.71684

**Published:** 2024-10-17

**Authors:** Leena Saeed, Gul Sharif, Sanjay Eda, Immanuel Raju Tullimalli, Adil Amin, Abdallah A Riyalat, Fauwaz F Alrashid, Alaa A Abdelrahim

**Affiliations:** 1 Medical Research Center, Hamad Medical Corporation, Doha, QAT; 2 General Surgery, Peshawar Reading Hospital, Peshawar, PAK; 3 Medicine, Manthena Narayana Raju (MNR) Medical College and Hospital, Fasalwadi, IND; 4 Medicine, Guangxi Medical University, Guangxi, CHN; 5 Cardiology, Pakistan Navy Station (PNS) Shifa, Karachi, PAK; 6 Internal Medicine, Sidra Medicine, Doha, QAT; 7 Surgery, College of Medicine, University of Hail, Hail, SAU; 8 Internal Medicine, Hamad Medical Corporation, Doha, QAT

**Keywords:** bariatric metabolic surgery, cardiovascular outcomes, glp-1 receptor agonists, meta-analysis, mortality

## Abstract

Cardiovascular disease (CVD) remains a leading cause of morbidity and mortality worldwide, particularly in individuals with obesity and type 2 diabetes mellitus (T2DM). This meta-analysis aimed to compare the effectiveness of bariatric metabolic surgery (BMS) and glucagon-like peptide-1 receptor agonists (GLP-1RAs) on cardiovascular outcomes and mortality in patients with obesity. A comprehensive literature search was conducted in Medline, Embase, and Cochrane CENTRAL from inception to September 15, 2024. Four observational studies meeting the inclusion criteria were analyzed, comprising a total of 247,000 patients. The primary outcomes were major adverse cardiovascular events (MACE) and all-cause mortality. Random effects models were used to calculate pooled risk ratios (RR) with 95% confidence intervals (CI). The results showed that BMS was associated with a significantly lower risk of MACE compared to GLP-1RAs (RR: 0.71, 95% CI: 0.56-0.90, p = 0.004), indicating a 29% reduction in MACE risk. Additionally, BMS demonstrated a 25% reduction in all-cause mortality risk (RR: 0.75, 95% CI: 0.65-0.87, p < 0.0001). These findings suggest that BMS offers superior cardiovascular protection and improved survival outcomes compared to GLP-1RAs in obese patients. The observed benefits may be attributed to more significant reductions in body mass index (BMI) and improvements in metabolic parameters achieved through surgical intervention. However, the limitations of this study, including its observational nature and the limited number of included studies, underscore the need for further research, particularly randomized controlled trials (RCTs), to confirm these findings and guide clinical decision-making in obesity management.

## Introduction and background

Cardiovascular disease (CVD) remains the leading cause of morbidity and mortality worldwide, particularly in individuals with obesity and associated metabolic conditions such as type 2 diabetes mellitus (T2DM) [1,2]. The complex interplay between obesity, insulin resistance, and cardiovascular health has made the identification of effective long-term treatments crucial for reducing cardiovascular risks and improving survival rates [3]. Bariatric metabolic surgery (BMS) and glucagon-like peptide-1 receptor agonists (GLP-1RAs) represent two prominent therapeutic approaches for obesity and its cardiovascular complications [4,5]. However, the comparative impact of these interventions on cardiovascular outcomes and mortality remains a critical question.

Bariatric metabolic surgery, encompassing procedures such as Roux-en-Y gastric bypass (RYGB) and sleeve gastrectomy, has been consistently linked to significant reductions in body weight, improvements in metabolic parameters, and remission of comorbidities such as T2DM and hypertension [6,7]. In terms of cardiovascular outcomes, several observational studies and clinical trials have demonstrated a reduction in major adverse cardiovascular events (MACE), including myocardial infarction and stroke, as well as lower overall mortality in patients who undergo BMS [8]. These benefits are thought to arise from a combination of factors, including profound weight loss, improved insulin sensitivity, and favorable changes in lipid profiles and inflammatory markers [9].

Liraglutide and semaglutide, two GLP-1RAs, on the other hand, provide a pharmaceutical option for controlling obesity and the risk of CVD. GLP-1RAs are powerful incretin mimetics that lower blood pressure, increase endothelial function, and enhance glycemic management in addition to helping people lose weight [10,11]. Regardless of prior myocardial infarction, it has been observed that both ligandiputra and dulaglutide can reduce weight and lessen the risk of MACE in patients with T2D and CVD by 12%-13% [12]. Similarly, semaglutide has been shown to reduce MACE risk in individuals with and without T2DM by 20%-26% when compared to placebo [13].

Notably, large cardiovascular outcome trials (CVOTs) have shown that GLP-1RAs reduce the incidence of MACE and cardiovascular mortality in patients with T2DM and high cardiovascular risk [14]. These drugs are especially appealing for patients seeking non-surgical, medically managed interventions with a favorable safety profile. Despite the demonstrated efficacy of both BMS and GLP-1RAs in reducing cardiovascular risks, there is limited direct comparative evidence between the two treatment modalities regarding long-term cardiovascular outcomes and mortality [15]. The surgical nature of BMS offers the potential for more profound and durable metabolic improvements, but it is also associated with perioperative risks and long-term complications. On the other hand, GLP-1RAs are associated with lower procedural risks but may not achieve the same magnitude of weight loss and metabolic change [16,17].

This meta-analysis seeks to systematically evaluate the cardiovascular outcomes and mortality associated with BMS compared to GLP-1RAs. By synthesizing the latest clinical evidence, this study aims to provide a clearer understanding of the relative benefits and risks of these interventions, offering valuable insights for clinicians in tailoring cardiovascular risk management strategies for patients with obesity.

## Review

Methodology

This meta-analysis follows the Preferred Reporting Items for Systematic Reviews and Meta-Analyses (PRISMA) guidelines and adheres to the principles outlined in the Cochrane Handbook and Meta-analysis Of Observational Studies in Epidemiology (MOOSE) guidelines.

Search Strategy

A comprehensive literature search was conducted in Medline (via PubMed), Embase, and the Cochrane Central Register of Controlled Trials (CENTRAL) from inception to September 15, 2024. The search identified studies comparing cardiovascular outcomes and mortality between bariatric metabolic surgery (BMS) and glucagon-like peptide-1 receptor agonists (GLP-1RAs). Keywords used to search for relevant articles included "glucagon-like peptide-1 receptor agonists" OR "GLP-1RAs" AND "bariatric metabolic surgery" OR "BMS" AND "all-cause mortality" OR "death" OR "cardiovascular events" OR "cardiovascular outcomes." Besides this, Medical Subject Heading (MeSH) terms were used to optimize the search. Additional searches were conducted in ClinicalTrials.gov and the International Clinical Trials Registry Platform (ICTRP) for unpublished or ongoing trials. To ensure thoroughness, reference lists of relevant reviews and included studies were screened for potential missed studies. The search was conducted without language restrictions, and authors of the included studies were contacted to obtain any unpublished data if necessary.

Study Selection

Two independent reviewers screened titles and abstracts of retrieved articles using EndNote software (Clarivate, London, UK) for deduplication. Studies that potentially meet the inclusion criteria were proceeded to full-text screening. Eligible studies involved adult patients with obesity who underwent either bariatric metabolic surgery or received GLP-1RA therapy and reported cardiovascular outcomes such as myocardial infarction, stroke, cardiovascular mortality, or a composite of all these or overall mortality. Disagreements during study selection were resolved by discussion or by consulting a third reviewer. Cohort studies and randomized controlled trials were included in this meta-analysis. We excluded case reports, case series, reviews, and editorials. We also excluded studies lacking control or comparison groups. We also excluded studies that did not report any of the outcomes assessed in this meta-analysis.

Data Extraction

Two reviewers used a standard extraction form in Microsoft Excel (Microsoft Corp., Redmond, WA) to extract the data separately. Study characteristics (author names, publication year, country, and study design) and duration of follow-up were among the extracted data. Cardiovascular events and all-cause mortality were included in the outcome measures.

Quality Assessment

For cohort and case-control studies, the Newcastle-Ottawa Scale (NOS) was used to evaluate the quality of the included research. Three primary domains were assessed by the NOS: the choice of study groups, group comparability, and identification of the desired outcome or exposure. A maximum of nine points might be awarded to each study, with two points going toward comparability, three points for outcome evaluation, and up to four points for selection. Studies that received seven or more points were rated as high quality, studies that received five to six points were rated as moderate quality, and studies that received less than five points were rated as low quality. The included studies were evaluated by two separate reviewers, and any disagreements in the scores were settled by discussion or by contacting a third reviewer.

Data Analysis

Data analysis was conducted using Review Manager (RevMan) software (The Cochrane Collaboration, London, UK). Risk ratios (RR) were pooled for both cardiovascular outcomes and mortality along with a 95% confidence interval (CI) using a random effects model to account for between-study heterogeneity. A p-value of less than 0.05 was considered significant. The I² statistic was calculated to evaluate heterogeneity, with I² values above 50% indicating significant heterogeneity. Forest plots were presented for pooled analyses. Publication bias was not assessed because of the lack of a sufficient number of studies.

Results

**Figure 1:**
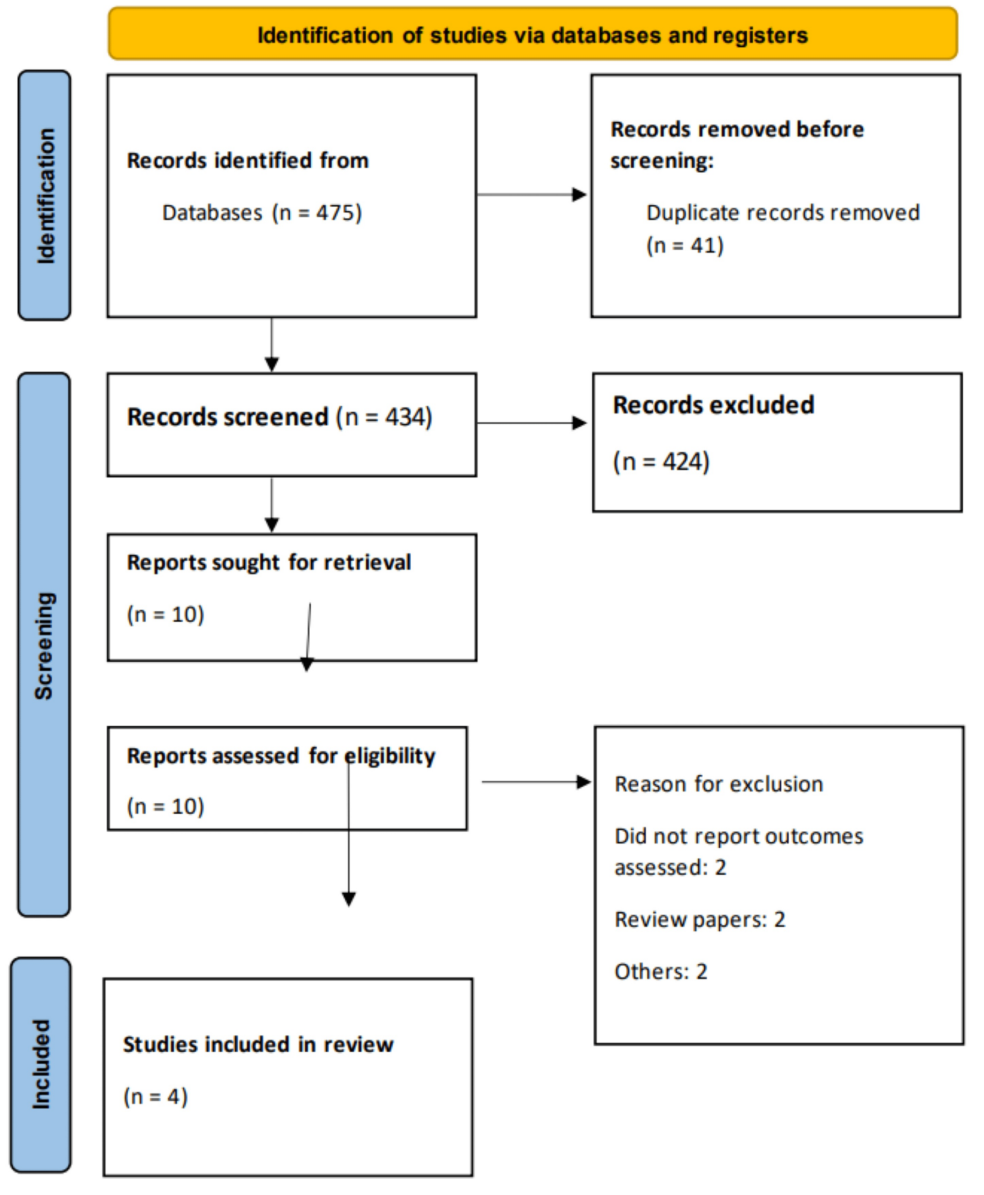
PRISMA flowchart PRISMA: Preferred Reporting Items for Systematic Reviews and Meta-Analyses

The initial database search yielded a total of 475 records from Medline, Embase, and Cochrane CENTRAL. After removing duplicates, 434 studies remained for the title and abstract screening. Of these, 424 studies were excluded based on irrelevance to the research question or failure to meet inclusion criteria. A total of 10 full-text articles were then assessed for eligibility. After a thorough evaluation, six studies were excluded. Ultimately, four studies were included in the meta-analysis. The study selection process is summarized in the PRISMA flow diagram (Figure 1). Table 1 presents the characteristics of the included studies. Table 1 summarizes four observational studies comparing cardiovascular outcomes between bariatric metabolic surgery and GLP-1RAs [18-21]. The studies, published between 2023 and 2024, encompass diverse regions including Sweden, Israel, and the United States. Follow-up durations ranged from 4.2 to 10 years, allowing for long-term observation of outcomes. The total population included in these studies was substantial, with a combined sample size of over 247,000 patients. Propensity score matching (PSM) was employed in most studies, except for one by Dicker et al. [19], which used no PSM. Table 2 presents the quality assessment of the included studies [18-21].

**Table 1:** Characteristics of the included studies PSM: propensity score matching, GLP-1RA: glucagon-like peptide-1 receptor agonist

Author	Year	Design	Analysis	Region	Follow-up duration	Study groups	Sample size
Adekolu et al. [18]	2023	Observational	PSM	United States	10 years	Surgery	118,828
						GLP-1RA group	118,828
Dicker et al. [19]	2024	Observational	No PSM	Israel	6.8 years	Surgery	3,035
						GLP-1RA group	3,035
Stenberg et al. [20]	2023	Observational	PSM	Sweden	4.2 years	Surgery	2,161
						GLP-1RA group	2,161
Stenberg et al. [21]	2024	Observational	PSM	Sweden	7 years	Surgery	2,039
						GLP-1RA group	2,039

**Table 2:** Quality assessment of the included studies

Author	Selection	Comparison	Assessment	Overall
Adekolu et al. [18]	++	+	++	Fair
Dicker et al. [19]	++++	++	+++	Good
Stenberg et al. [20]	+++	++	++	Good
Stenberg et al. [21]	++++	++	+++	Good

Comparison of Cardiovascular Events Between Surgery and GLP-1RAs

**Figure 2:**
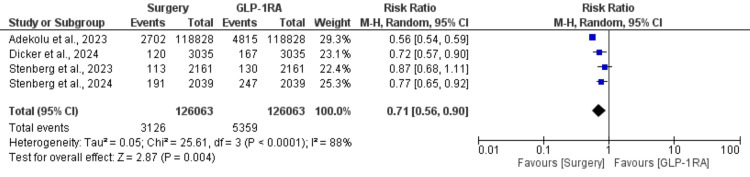
Comparison of cardiovascular events between surgery and GLP-1RAs References: Adekolu et al. [18], Dicker et al. [19], Stenberg et al. [20], and Stenberg et al. [21] GLP-1RAs: glucagon-like peptide-1 receptor agonists

The meta-analysis included four observational studies comparing the incidence of major adverse cardiovascular events (MACE) between bariatric metabolic surgery and GLP-1RAs, with a total of 120,063 patients in each group. Bariatric surgery was associated with a significantly lower risk of MACE compared to GLP-1RAs, with a pooled risk ratio (RR) of 0.71 (95% CI: 0.56-0.90, p = 0.004) (Figure 2). This indicates a 29% reduction in MACE risk for patients undergoing bariatric surgery. Heterogeneity among studies was significant (I² = 88%, p < 0.0001) (Figure 2), suggesting variability in effect sizes across studies. Despite this, the overall effect favored bariatric surgery in reducing cardiovascular events.

Comparison of All-Cause Mortality Between Surgery and GLP-1RAs

**Figure 3:**
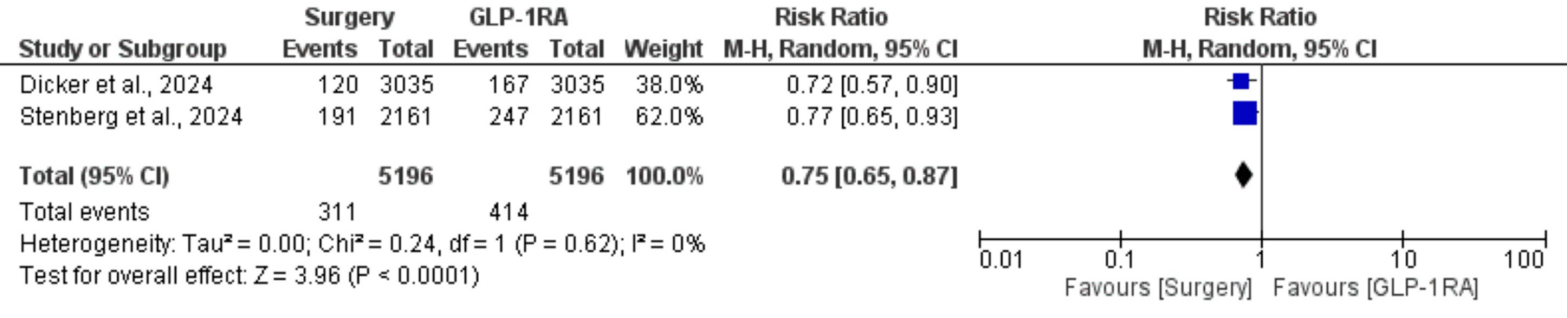
Comparison of all-cause mortality between surgery and GLP-1RAs References: Dicker et al. [19] and Stenberg et al. [21] GLP-1RAs: glucagon-like peptide-1 receptor agonists

Two studies assessed all-cause mortality, comparing bariatric metabolic surgery to GLP-1RAs, with a total of 5,196 patients in each group. The meta-analysis showed a significant reduction in all-cause mortality for patients who underwent bariatric surgery, with a pooled risk ratio (RR) of 0.75 (95% CI: 0.65-0.87, p < 0.0001) (Figure 3). There was no significant heterogeneity between the studies (I² = 0%, p = 0.62), indicating consistent results across studies (Figure 3). This finding suggests that bariatric surgery is associated with a 25% reduction in the risk of all-cause mortality compared to GLP-1RAs.

Discussion 

This meta-analysis provides robust evidence that bariatric metabolic surgery significantly reduces both major adverse cardiovascular events (MACE) and all-cause mortality compared to glucagon-like peptide-1 receptor agonists (GLP-1RAs) in patients with obesity. Our findings suggest that bariatric surgery offers superior cardiovascular protection, with a 29% reduction in MACE and a 25% reduction in all-cause mortality, highlighting its potential as an effective intervention for long-term cardiovascular risk reduction. These results are consistent with previous research demonstrating the cardiovascular benefits of weight loss achieved through surgical interventions, which extend beyond glycemic control and weight reduction [22].

The meta-analysis performed by Syn et al. reported reduced rates of death among patients treated with BMS compared to patients treated with non-surgery. However, the study reported that the reduction in the risk of death was higher in individuals with diabetes compared to their counterparts [23]. We were not able to compare the benefits of two treatment options separately in diabetes and non-diabetes patients due to the limited number of studies. The survival benefit linked to bariatric metabolic surgery (BMS) in contrast to treatment with GLP-1RAs may be attributed to the more significant relative reduction in body mass index (BMI) seen in patients who underwent BMS compared to those treated with GLP-1RAs [19,20]. Aminian et al. found a correlation between BMS and decreased mortality over an average follow-up period of 4.9 years [24]. Notably, while at least a 5% weight loss was necessary to lower the risk of all-cause mortality in diabetic patients who had BMS, a reduction of 20% in weight loss was required for those who did not undergo the procedure [25].

MACE represent a primary source of illness and death among obese individuals with diabetes mellitus [26]. Current clinical guidelines for DM management stress the importance of reducing MACE risk factors through improved glycemic control, blood pressure management, and lipid level optimization [27]. Despite intensive lifestyle changes and medication, many patients fail to meet these recommended targets, leading to ongoing health issues and increased healthcare costs [27]. Our findings suggest that BMS is linked to a notable reduction in morbidity and mortality from MACE when compared to treatment with GLP-1RAs.

An examination of data from various studies on GLP-1RAs has shown their positive effects in type 2 diabetes patients. These treatments were associated with decreased incidence of major adverse cardiovascular events (MACE) and lower overall mortality when compared to placebo groups [28,29]. Research indicates notable reductions in cardiovascular incidents, including myocardial infarction, stroke, and deaths from cardiovascular causes or any cause, with improvement rates between 9% and 16% post-treatment [30]. GLP-1RAs are thought to provide cardiovascular benefits by influencing several risk factors. These include promoting weight loss, reducing blood pressure, decreasing levels of low-density lipoprotein cholesterol and triglycerides, and improving glycemic control. These combined effects may contribute to the observed reduction in MACE [31]. Interestingly, bariatric metabolic surgery (BMS) impacts these same factors, but to a greater extent. This study revealed that BMS patients experienced more significant weight reduction, better blood sugar management, and a higher proportion of reaching target lipid levels. Additionally, BMS has been found to increase postprandial GLP-1 plasma concentrations [32]. This effect, combined with the more pronounced improvements in cardiovascular risk factors, may explain why BMS appears to have a greater impact on reducing MACE compared to GLP-1RA treatments alone. The comprehensive physiological changes induced by BMS suggest that it could offer a more potent approach to addressing cardiovascular risk in type 2 diabetes patients.

The present meta-analysis has certain limitations. Firstly, only four studies were included in this meta-analysis. All studies were observational in nature. There is an RCT comparing BMS and GLP-1RAS. Secondly, we were not able to perform subgroup analysis based on certain factors such as the presence of diabetes and Hb1AC level as these factors are likely to impact the outcomes. Thirdly, we did not assess individual cardiovascular outcomes such as myocardial infarction, heart failure, and cardiovascular death as no studies assessed these individual outcomes. Additionally, we were not able to perform an analysis based on the type of GLP-1RA used. We need further trials directly comparing bariatric surgery and GLP-1RA for proper planning and counseling for individuals with obesity. This evaluation gives clinicians more confidence to recommend bariatric surgery since, despite powerful new weight-lowering drugs, it is still an underutilized therapeutic option that still results in the greatest weight loss.

## Conclusions

This meta-analysis provides compelling evidence that bariatric metabolic surgery (BMS) offers superior cardiovascular protection compared to glucagon-like peptide-1 receptor agonists (GLP-1RAs) in patients with obesity. The findings demonstrate a significant reduction in major adverse cardiovascular events (MACE) and a decrease in all-cause mortality associated with BMS. These results underscore the potential of BMS as a more effective intervention for long-term cardiovascular risk reduction and improved survival outcomes. The observed benefits likely stem from the more pronounced improvements in body mass index, glycemic control, and overall metabolic profile achieved through surgical intervention. While GLP-1RAs remain a valuable non-surgical option, BMS appears to offer more substantial and durable metabolic improvements. However, the limitations of this study, including its observational nature and limited number of included studies, highlight the need for further research, particularly randomized controlled trials, to confirm these findings and guide clinical decision-making in obesity management.
